# Predicting response to non-selective beta-blockers with liver–spleen stiffness and heart rate in patients with liver cirrhosis and high-risk varices

**DOI:** 10.1007/s12072-024-10649-7

**Published:** 2024-04-25

**Authors:** Mauro Giuffrè, Johannes Dupont, Alessia Visintin, Flora Masutti, Fabio Monica, Kisung You, Dennis L. Shung, Lory Saveria Crocè, The NSBB-Elasto-Response-Prediction Group, Mauro Giuffrè, Johannes Dupont, Alessia Visintin, Flora Masutti, Fabio Monica, Kisung You, Lory Saveria Crocè, Cristiana Abazia, Clara Faini, Michele Campigotto, Francesca Dottor, Marco Gulotta, Irma Valeria Albergati, Dennis L. Shung

**Affiliations:** 1 Department of Internal Medicine (Digestive Diseases), Yale School of Medicine, Yale University, New Haven, CT USA; 2 Department of Medical, Surgical and Health Sciences, University of Trieste, Trieste, Italy; 3 Faculty of Medicine, University of Cologne, Cologne, Germany; 4 Liver Clinic, Trieste University Hospital, Trieste, Italy; 5 Gastroenterology and Endoscopy Unit, Trieste University Hospital, Trieste, Italy; 6 Barauch College, Department of Mathematics, City University of New York, New York, NY USA

**Keywords:** Liver cirrhosis, High-risk varices, Non-selective beta-blockers (NSBB), Primary prophylaxis, Spleen stiffness (SS), Liver stiffness (LS), Hepatic venous pressure gradient (HVPG), Elastography, Variceal hemorrhage, Machine learning

## Abstract

**Introduction:**

Non-selective beta-blockers (NSBB) are used for primary prophylaxis in patients with liver cirrhosis and high-risk varices (HRVs). Assessing therapeutic response is challenging due to the invasive nature of hepatic venous pressure gradient (HVPG) measurement. This study aims to define a noninvasive machine-learning based approach to determine response to NSBB in patients with liver cirrhosis and HRVs.

**Methods:**

We conducted a prospective study on a cohort of cirrhotic patients with documented HRVs receiving NSBB treatment. Patients were followed-up with clinical and elastography appointments at 3, 6, and 12 months after NSBB treatment initiation. NSBB response was defined as stationary or downstaging variceal grading at the 12-month esophagogastroduodenoscopy (EGD). In contrast, non-response was defined as upstaging variceal grading at the 12-month EGD or at least one variceal hemorrhage episode during the 12-month follow-up. We chose cut-off values for univariate and multivariate model with 100% specificity.

**Results:**

According to least absolute shrinkage and selection operator (LASSO) regression, spleen stiffness (SS) and liver stiffness (LS) percentual decrease, along with changes in heart rate (HR) at 3 months were the most significant predictors of NSBB response. A decrease > 11.5% in SS, > 16.8% in LS, and > 25.3% in HR was associated with better prediction of clinical response to NSBB. SS percentual decrease showed the highest accuracy (86.4%) with high sensitivity (78.8%) when compared to LS and HR. The multivariate model incorporating SS, LS, and HR showed the highest discrimination and calibration metrics (AUROC = 0.96), with the optimal cut-off of 0.90 (sensitivity 94.2%, specificity 100%, PPV 95.7%, NPV 100%, accuracy 97.5%).

## Introduction

Portal hypertension (PH) is a significant complication in patients with chronic advanced liver disease, leading to the development of esophageal varices (EVs), ascites, hepatic encephalopathy (HE), and hepatorenal syndrome (HRS), which contribute to increased morbidity and mortality [1]. The gold-standard method for diagnosing clinically significant portal hypertension (CSPH) is hepatic venous pressure gradient (HVPG), an invasive and expensive procedure available only in specialized centers [2]. Therefore, non-invasive methods have been investigated to serve as surrogates of HVPG measurement to stage PH [3–5]. Despite the development and validation of several laboratory values-based scores, liver stiffness (LS) has emerged as a non-invasive, reliable, and widely accepted method for assessing the degree of liver fibrosis, cirrhosis, and stratification of PH, thus serving as a non-invasive marker for  liver disease management and risk-stratification [3]. LS has been widely adopted and validated as an EVs screening surrogate, with the Baveno VI guidelines confidently recommending that compensated cirrhotic patients with LS < 20 kPa and platelets > 150,000/mm^3^ had a 95% possibility for not developing high-risk varices (HRVs) [6]. During the transition period between previous and current guidelines, spleen stiffness (SS) has emerged as a comparable technique, if not superior, to LS in staging and risk prediction in patients with CSPH [7, 8]. In fact, according to Baveno VII recommendations, elastography is sufficiently accurate to identify CSPH in clinical practice [9]: in non-obese patients with compensated advanced chronic liver disease (cACLD), LS ≥ 25 kPa is sufficient to rule-in CSPH (specificity and positive predictive value > 90%). In addition, SS cutoff has been introduced to rule-in CSPH for viral-related cirrhosis (SS ≥ 50 kPa) and to identify those patients at low probability of HRVs that are required to undergo EGD according to Baveno VI criteria, in whom endoscopy can be avoided (SS ≤ 40 kPa) [9]. Implementing an elastography-driven definition of CSPH has also had significant therapeutic implications, particularly regarding indications for non-selective beta-blocker (NSBB) therapy. According to the new Baveno VII statements, the decision to treat with NSBB should be independent of HVPG measurements, and patients currently on NSBB are not required to undergo screening EGD for the detection of EVs [9]. However, it is still unclear how to monitor NSBB hemodynamic response without HVPG measurements, especially considering that the number needed to treat (NNT) in primary prophylaxis ranges between 5 and 13 [10]. The non-invasive strategy to screen and treat patients with CSPH carries the risk of being unable to monitor the efficacy of NSBB fully, thus exposing non-responders to an increased risk of variceal hemorrhage.

There are few studies that explored the role of liver and spleen elastography on the correlation between SS or LS values with HVPG measurements at baseline and in response to NSBB treatment [11–14]. According to two studies, SS appears to be the best predictor of hemodynamic response to NSBB [13, 14], and a percentual decrease of at least 10% from individual values is highly predictive of response [13]. On the contrary, *Binzberger *et al*.* [12] demonstrated that neither LS nor SS could reliably predict response to NSBB. These preliminary findings need to be further validated and explored in other clinical settings, in order to define if elastography can be used to monitor response to NSBB. 

Thus this study aims to create a machine-learning algorithm to predict non-invasively response to NSBB in patients with HRVs on primary prophylaxis for variceal hemorrhage who were undergoing serial measurements of LS and SS in the first 12 months following NSBB administration.

## Materials and methods

### Study design, patient follow-up, and data collection

The present study is a prospective observational cohort study conducted at a single center (Trieste University Hospital) enrolling patients referred to the Liver Clinic Unit. The study consisted of two parts: the first part (1st May 2018 to 31st December 2020) aimed to derive a prediction model, whereas the second part (1st January 2021 to 31st December 2021) was designed to enroll a cohort of patients where the model could be validated. We enrolled consecutive patients with a diagnosis of liver cirrhosis and the presence of HRVs and an indication for primary prophylaxis with NSBB for first variceal bleeding prevention. The diagnosis of liver cirrhosis was established utilizing a combination of clinical, biochemical, and ultrasound imaging (e.g., nodular liver surface, coarse liver echotexture), and/or histological examination [15, 16]. The prescription and initiation of NSBB therapy were part of the patient's routine therapeutic course, and their participation in the study did not interfere with the established practice of treatment, which was assessed following current guidelines.

**Fig. 1:**
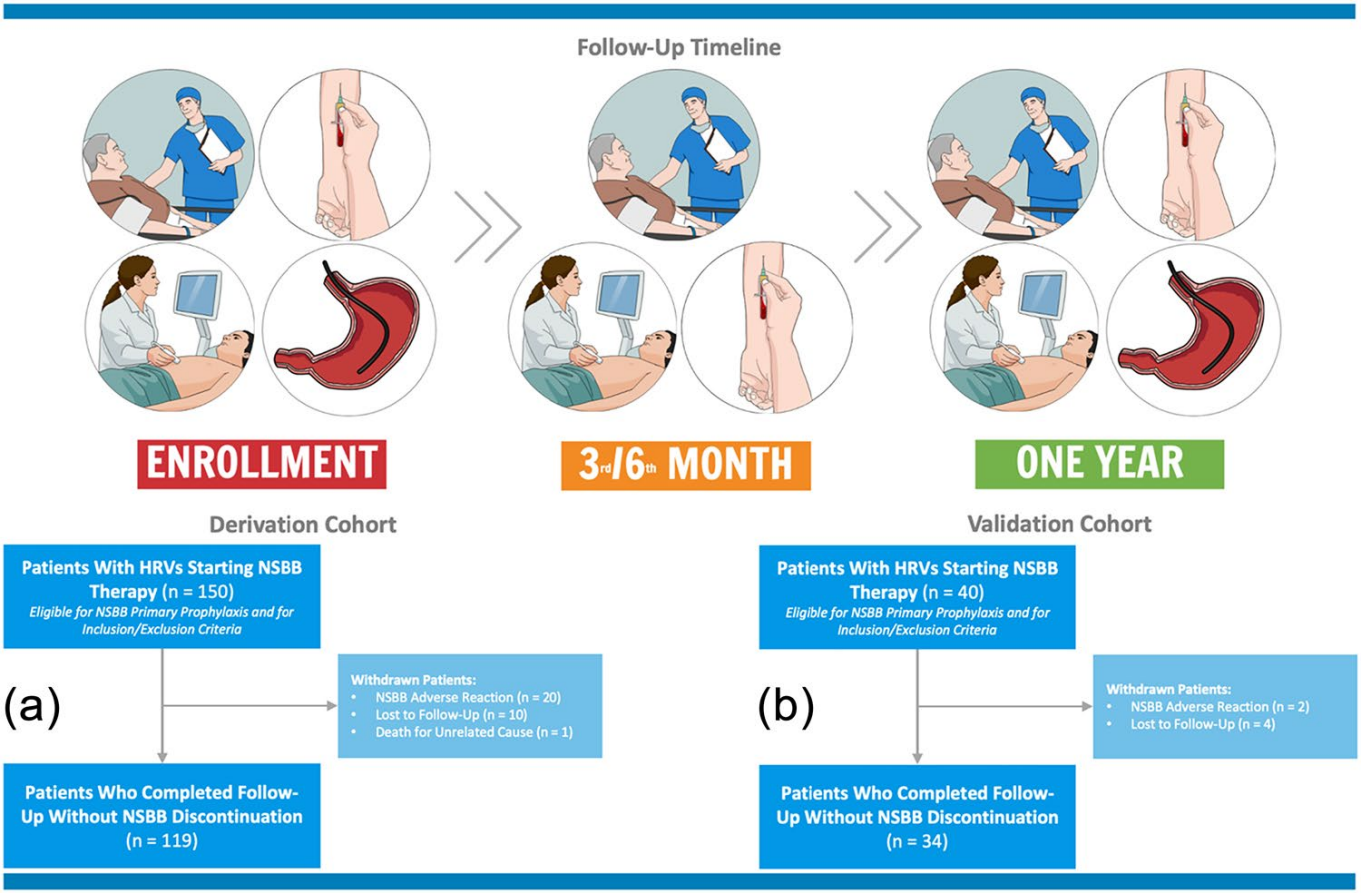
**Description of follow-up timeline from enrollment.** Eligible patients underwent baseline clinical assessment, laboratory tests, and elastography measurements liver stiffness > 20 kPa and platelet count < 150.000 × 10^9^ cells/L underwent EGD for EVs screening within 10 days. Patients with HRVs were prescribed NSBBs, and re-evaluated at 3/6 months, and 1-year post-therapy initiation. **a** Flowchart reporting patients who completed follow-up in the derivation cohort, while **b** reports patients who completed follow-up in the validation cohort

Each eligible patient was evaluated at baseline through clinical assessment (physical examination, vital parameters such as heart rate and arterial blood pressure, and anthropometric characteristics such as height, weight, and calculation of body mass index) and with the following laboratory tests: white blood cell count (WBC), hematocrit, hemoglobin, platelet count, INR, aspartate aminotransferase (AST), alanine aminotransferase (ALT), gamma-glutamyl transferase (GGT), alkaline phosphatase (ALP), sodium, potassium, total bilirubin, albumin, and creatinine. The Child–Pugh [17] and model for end-stage liver disease (MELD) [18] scores were calculated for each patient. On the same day, each patient underwent liver and spleen elastography measurements and evaluation of ultrasonographic parameters such as portal vein diameter, portal flow velocity, spleen bipolar diameter, and spleen surface measured at the hilum. After this initial screening, patients with LS > 20 kPa and platelet count < 150.000 × 10^9^ cells/L underwent EGD for EVs screening within 10 days. Patients with endoscopic evidence of HRVs and without NSBB contraindications were prescribed an NSBB with dose titration as suggested by the American Association of the Study of the Liver guidelines [1]. Propranolol was started with a dose of 20–40 mg orally twice a day, with dose adjusting every 2–3 days (maximum daily doses of 320 mg/day in patients without ascites and 160 mg/day in patients with ascites). Carvedilol was started with a dose of 6.25 mg once a day and a dose adjustment every 3 days (maximum daily dose of 12.5 mg/day). Each patient who started NSBB therapy was closely monitored in the first 3 weeks for dose titration and then re-evaluated after 3 months (physical examination, liver, and spleen elastography, and laboratory exams), six (physical examination, liver, and spleen elastography, liver ultrasound examination, and laboratory exams), and twelve (physical examination, liver, and spleen elastography, liver ultrasound examination, laboratory exams, and EGD) months after therapy initiation, as shown in Fig. 1.

### Inclusion criteria

The inclusion criteria for the study were: age > 18 years with liver cirrhosis regardless of etiology, who had undergone esophagogastroduodenoscopy (EGD) according to Baveno VI criteria (LS > 20 kPa and platelet count < 150.000 × 10^9^ cells/L) [19] with evidence of HRVs and candidate to NSBB therapy.

### Exclusion criteria

We categorically excluded patients with previously diagnosed and/or treated for hepatocellular carcinoma (HCC), portal vein thrombosis, previous treatment with NSBB or endoscopic variceal band ligation (EBL), with contraindications to NSBB administration (heart rate < 50 bpm, systolic blood pressure < 100 mmHg, aortic disease, atrioventricular blocks, severe peripheral angiopathy, asthma or chronic obstructive pulmonary disease of any severity), pregnant female patients, severe obesity (BMI > 40), and known hematological disease of any kind. Furthermore, considering that ongoing liver injury, as delineated by guidelines, typically influences subsequent endoscopic evaluations for patients with suspected clinically significant portal hypertension (CSPH) [1] and may impact NSBB response prediction, we chose to exclude all patients with ongoing liver injury (e.g., ongoing alcohol abuse, untreated HCV or HBV infection, or autoimmune flares without immunosuppressive treatment). In addition, due to limited resources, we made the decision to exclude from the final analysis any patients who experienced variceal hemorrhage prior to their initial elastography follow-up after beginning NSBB treatment (i.e., 3 months after first administration).

### Endoscopic assessment of esophageal varices

EGD was performed by the staff of the Gastrointestinal and Endoscopy Service at Trieste University Hospital using PENTAX endoscopic devices EPK-i7010 series. All the instruments are at high definition. The exploration of the esophagus was performed spending at least 3 min initially in a deflated state, obtaining at least three images (upper, middle, and lower esophagus), and then at maximum insufflation using CO2, again obtaining at least three images (upper, middle, and lower esophagus). The images obtained were stored in the EndoxWeb software available in the hospital management system. The endoscopic classification of EVs was performed according to the Beppu Classification [20]. HRVs were defined as EVs ≥ F2 with/without red signs or F1 with red signs. For each EGD, EVs classification was assessed by a second experienced endoscopist, blind to the first endoscopist grading, using the stored image obtained by the endoscopist who performed the EGD. When agreement was not reached between the two classifiers, a third endoscopist was consulted to confirm one of the two classifications. All the endoscopists are experts in EVs diagnostic and therapeutic evaluation both in elective and urgent settings.

### Outcome definition

We defined a binary outcome of response to NSBB according to endoscopic evaluation or the presence of variceal hemorrhage. We defined as “responders” the patients with stationary or downstaged variceal grading at the 12-month EGD. In contrast, we defined as “non-responders” the patients with upstaging variceal grading at the 12-month EGD or at least one variceal hemorrhage episode during the 12-month follow-up.

### Elastography measurement

Liver and Spleen Stiffness were measured using a Philips Affiniti 70 (ElastPQ Protocol) ultrasonography system with a 1–5 MHz convex probe [21–26]. All measurements were performed by four experienced operators (> 500 elastography examinations each). Patients were positioned in supine decubitus with the right arm (liver) or left arm (spleen) in maximal abduction to increase the intercostal acoustic window. The region of interest (ROI) was placed between the VII and VIII segments at least 1.5 cm from the hepatic capsule (LS) and at the splenic hilum or lower pole at least 1 cm from the splenic capsule (SS) [22]. The ROI was accurately located in an area without large liver vessels, bile ducts, and rib shadows. During the acquisition, the patient was requested to hold his/her breath for 5 s [27]. All measures obtained after a deep inspiration, maximal expirations, and Valsalva maneuver were discarded. In some cases, breath-hold was practiced with the patient prior to initiating elastography. Ten different valid elastography measurements were obtained in all subjects, both in the liver and the spleen, and the median value was used. The measure obtained was acquired only if its standard deviation was < 30% [27]. We defined “technical failure” as the impossibility of obtaining any value or an IQR/M ≥ 0.30 and selected values with an IQR/M < 0.30 [27]. All patients were examined after overnight fasting and without caffeine intake in the previous 3 h. Each physician performing elastography examination was blind to the initial endoscopic patient status and was not informed about the initial endoscopic status or development of complications (e.g., variceal hemorrhage) in any of the follow-up appointments.

### Adaptation of Kim et al.’s model

*Kim *et al*.* [14] proposed a univariate model based on SS. In particular, the linear predictor (LP) was calculated as follows: 0.0490–2.8345 × ΔSS, where ΔSS was defined as SS (3rd-Month-Follow-up) – SS(Enrollment). In addition, Kim et al. employed the Siemens Acuson S2000TM ultrasound system (Siemens AG, Erlangen, Germany) to perform LS and SS measurements and provided results in m/s. Given that our system provided measurements in kPa, they were converted into m/s using the following conversion: Measure_m/s_ = √(Measure_kPa_/3).

### Statistical analysis

According to our sample size, the Shapiro–Wilk test was performed to verify the normal distribution of variables [28], whose results indicated the absence of normally distributed variables, which were therefore reported as median (Quartile 1; Quartile 3). Differences between continuous variables were examined using the Mann–Whitney *U* test. Variable correlations were analyzed using the Spearman’s rank correlation test [29]. Exploratory data analysis in the derivation cohort revealed moderate to high correlations between variables, thus the need for variable shrinkage to avoid overfitting. Therefore, we employed the least absolute shrinkage and selection operator (LASSO) logistic regression [30] by selecting a penalization factor (i.e., lambda, λ) across tenfold cross-validation with the lowest cross-validation error, which resulted in a λ = 6.70. Then, we applied the penalization factor and selected variables with an absolute value of the β coefficient >|0.01|, which resulted in the selection of three variables: 3-month LS percentual decrease, 3-month SS percentual decrease, and 3-month HR percentual decrease. Univariate logistic regression was performed for each of the three variables with tenfold cross-validation, followed by multivariate logistic regression using all three variables. For each model, the primary performance measure was measured using the area under the receiver-operating characteristic curve (AUROC), and calibration with Nagelkerke Pseudo-R2, Akaike Information Criterion (AIC), Bayesian Information Criterion (BIC). Statistical comparison of the AUROC for the separate models was performed using DeLong’s Test. The model was developed to predict response to NSBB. Therefore, for cut-off analysis, it is crucial to identify those patients who do not respond to NSBB treatment. Achieving a high specificity is important for this group, because false positives must be very rare so that patients who do not respond to treatment will be closely monitored or sent to more invasive diagnostic follow-up tests. Therefore, we planned to use a cut-off value that achieved a specificity of 100% (or closest to 100% if none reached 100%) and to compare sensitivities using McNemar’s matched pairs test. Each derived model and cut-off values were then evaluated in the validation cohort by AUROC analysis. For all analyses, two-sided statistical significance was defined as *p* < 0.05 [22]. The model provided in our cohort of patients was compared to the model by Kim et al. [14] in terms of AUROC (DeLong’s Test), AIC, BIC, and Nagelkerke Pseudo-R2. Data were analyzed using Python (Version 3.11.2) using numpy, matplotlib, pandas, scipy and sklearn packages.

## Results

A total of 165 patients were identified as eligible to be enrolled in the derivation cohort, however, six patients were excluded for the presence of contraindications to NSBB, five patients were excluded for concurrent diagnosis of HCC, two patients were excluded for the detection of portal vein thrombosis, and two did not agree to participate in the study. Therefore, a total of 150 patients were enrolled in the derivation cohort (Fig. 1). Among these, twenty patients withdrew from the study due to NSBB adverse reactions, ten patients were lost to follow-up, and one patient died for unrelated causes, with 119 patients having completed the 12-month follow-up and being included in the study. For the validation cohort, 50 patients were identified as eligible. However, eight patients were excluded for contraindications to NSBB, and two patients were excluded for concomitant diagnosis of HCC. Therefore, a total of 40 patients were enrolled in the validation cohort (Fig. 1). Among these, two patients withdrew from the study due to NSBB adverse reactions, and four patients were lost to follow-up, with 34 patients having completed the 12-month follow-up and being included in the study. Patients' baseline characteristics and time-related changes of significant variables are reported in Tables 1 and 2, respectively.

**Table 1:** Clinical, biochemical and ultrasonographic characteristics of the enrolled population (*N* = 119)

Variable	Derivation cohort *N* = 119	Responders *N* = 76	Non-responders *N* = 43	*p* value	Validation cohort *N* = 34	Responders *N* = 22	Non-responders *N* = 12	*p* value
Age (years)	74.5 (70;78)	75 (71;78)	72 (69;77.5)	NS	76 (70;79)	74 (69;81)	76 (72;80)	NS
Gender, female (*n*, %)	34 (28.8%)	17 (22.7%)	17 (39.5%)	*p* = 0.046	10 (29.4%)	3 (13.6%)	7 (58.3%)	*p* = 0.006
Etiology (*n*, %)								
HCV	50 (42%)	32 (42.1%)	18 (41.9%)	NS	19 (55.9%)	10 (45.5%)	9 (75%)	NS
ALD	54 (45.4%)	35 (46.1%)	19 (44.2%)	NS	13 (38.2%)	11 (50%)	2 (16.7%)	NS
NAFLD	15 (12.6%)	9 (11.8%)	6 (13.9%)	NS	2 (5.9%)	1 (4.5%)	1 (8.3%)	NS
Laboratory test and scores								
AST (U/L)	70.5 (52;80)	69 (55;79)	77 (48.5;82.5)	*p* = 0.04	66 (53;80)	65 (52;78)	67 (54;82)	NS
ALT (U/L)	54 (45;73)	53 (44;70)	56 (50;74)	NS	54.5 (44;70)	54 (43;69)	55 (45;71)	NS
GGT (U/L)	103 (74;110)	106 (95;111)	77 (68;106)	*p* < 0.001	100 (75;115)	98 (74;114)	101 (76;116)	NS
ALP (U/L)	120 (90;140)	119 (91;139)	122 (92;142)	NS	101 (70;190)	100 (69;180)	(71;200)	NS
Total bilirubin (mg/dL)	1.2 (0.8;1.3)	1.19 (0.7;1.3)	1.3 (0.8;1.5)	NS	1.4 (1.1;1.7)	1.3 (1.0;1.6)	1.5 (1.2;1.8)	NS
Creatinine (mg/dL)	1.1 (0.9;1.3)	1.1 (0.8;1.2)	1.1 (0.7;1.3)	NS	0.90(0.80;1.50)	0.89 (0.79;1.40)	0.91 (0.81;1.60)	NS
Albumin (g/L)	3.1 (2.8;3.4)	3.0 (2.8;3.5)	3.3 (3.0;3.5)	NS	2.9 (2.7;3.3)	2.8 (2.6;3.2)	3.0 (2.8;3.4)	NS
Platelet count (10^9^ cells/L)	90 (80;135)	92 (84;137)	88 (78;134)	NS	148 (107;160)	147 (106;159)	149 (108;161)	NS
Child–Pugh score	6 (5;6)	6 (5;6)	6 (5:6)	NS	6 (5;6)	6 (5;6)	6 (5;6)	NS
MELD score	10 (8;11)	10 (8;11)	10 (8;11)	NS	10 (8;12)	10 (8;12)	10 (8;12)	NS
Ultrasound parameters								
Portal vein diameter (cm)	1.6 (1.3;1.7)	1.4 (1.3;1.7)	1.7 (1.5:1.8)	*p* = 0.001	1.6 (1.5;1.7)	1.7 (1.6;1.8)	1.5 (1.4;1.6)	NS
Portal vein flow velocity (cm/s)	18.6 (16.5;20)	19 (16.7;20.5)	18 (16.4:19.4)	NS	18.6 (15.7;19.5)	18.7 (15.8;20.3)	18 (15.6;19.4)	NS
Spleen bipolar diameter (cm)	14 (13;15)	13.1 (12.8;14.5)	14.8 (14.1:15.4)	*p* < 0.001	14.2 (13.1;14.9)	14.5 (13.2;15.1)	13.8 (13.2;15.8)	NS
Spleen surface (cm^2^)	47 (45:52)	40 (39.3;45.5)	49 (45;54)	*p* < 0.001	47.5 (45.7;51.6)	49.6 (45.8;51.7)	46.3 (43.6;54.5)	NS
Vital signs								
Systolic blood pressure (mmHg)	128 (117;143)	127 (115;140)	129 (114;145)	NS	119 (110;150)	118 (109;145)	120 (110;154)	NS
Diastolic blood pressure (mmHg)	81 (74;85)	80 (73;84)	82 (74;85)	NS	75 (70;90)	74 (71;92)	75 (69;91)	NS
NSBB medication (*n*, %)								
Propranolol	35 (29%)	19 (25.3%)	16 (37.2%)	NS	5 (14.7%)	3 (13.6%)	2 (16.7%)	NS
Carvedilol	84 (71%)	57 (74.7%)	27 (62.8%)	NS	29 (85.3%)	19 (86.4%)	10 (83.3%)	NS

Continuous variables are reported by median and interquartile ranges (Quartile 1; Quartile 3). Patients are stratified by their response to NSBB treatment according to outcome definition. Statistically significant differences are expressed by two-tailed *p* values

*NSBB* non-selective beta-blocker, *NS* not significant

**Table 2:** Liver/Spleen Stiffness and Heart Rate changes from baseline values

Variable	Derivation cohort *N* = 119	Responders *N* = 76	Non-responders *N* = 43	*p *value	Validation cohort *N* = 34	Responders *N* = 22	Non-responders *N* = 12	*p *value
LS (kPa)								
Enrollment	44 (39:46.7)	44 (39;46.5)	43 (38.5;47)	NS	46 (40;48.8)	46 (40;47.5)	44 (40.5;49.5)	NS
3 months	39.2 (34.9;42.9)	35.2 (30;38.5)	42.6 (37;7;43.9)	*p* = 0.012	41.4 (37.3:44.9)	41 (35;45.1)	42 (39.2;44.2)	*p* = 0.019
6 months	37.9 (33:41)	36 (30;38)	39.8 (36.7;42.4)	*p* = 0.015	39.9 (34.9;42.8)	37 (33.5;42.3)	40.7 (36.2;43.1)	*p* = 0.021
12 months	36.9 (31.8;40.5)	35.2 (29.4;38)	39 (34;42.1)	*p* = 0.021	39.4 (33.2;42.6)	35 (31.4;41.5)	39.7 (35.5;42.9)	*p* = 0.020
LS % decrease								
3 months	9.2 (3.2;16.8)	13.6 (6.3;20.5)	3.3 (2.6;8.4)	*p* < 0.001	7.4 (2.4:17.7)	10.2 (3.3;20.7)	6 (1.9;8.3)	*p* = 0.016
6 months	12.1 (7.9;20.4)	17.3 (9;25.3)	8.9 (7.8;11)	*p* < 0.001	12.3 (7.8;18.7)	13.1 (9.1;27)	11 (6.3;14.2)	*p* = 0.035
12 months	12.5 (8.3;20.5)	18.1 (10;26.4)	10.2 (8.3;14)	*p* < 0.001	15.3 (10.4;22.3)	21 (11.6;29.5)	11 (9;17.2)	*p* < 0.001
SS (kPa)								
Enrollment	53.4 (47;61.8)	55 (49.2;64.2)	53 (46;69)	NS	57 (53;64.8)	57 (53;64.5)	57 (52;64)	NS
3 months	48 (41.6;53.2)	44 (38;48)	49 (42;58)	*p* = 0.010	49.6 (44.3;52.7)	48 (43;51)	51.5 (46;55.6)	*p* = 0.023
6 months	46 (39.8;50.9)	42.7 (37.6;46)	48 (41;57)	*p* = 0.021	46 (40.5;50.7)	43.6 (39.3;48)	49.9 (41.9;51.8)	*p* = 0.025
12 months	44.2 (38;50.2)	40 (38.6;45)	45 (39.7;52)	*p* = 0.029	45.6 (39.2;51.7)	44.6 (38.8;48)	50.3 (41.1;52.6)	*p* = 0.031
SS % decrease								
3 months	9.8 (6.5;13)	12.5 (11:15.5)	5.9 (5;7.5)	*p* < 0.001	14.3 (10.3;19.3)	16.9 (13.9;20.9)	10.2 (8.6;14.2)	*p* < 0.001
6 months	13.5 (10.8;16.3)	15.6 (13.2;19)	10.5 (7.8;13)	*p* < 0.001	19.2 (17.2;22.8)	22.4 (19.2;24.6)	17.2 (13;18.4)	*p* < 0.001
12 months	14.7 (10.9;18.1)	16.9 (13.5;22)	12 (8.9;14)	*p* < 0.001	19.2 (16.3;22)	20.7 (18.9;26.2)	17.2 (13.5;18.9)	*p* < 0.001
HR (bpm)								
Enrollment	82 (80;86)	82 (80;84)	83 (81;86)	NS	84 (82;87)	83 (81;87)	84 (83;87)	NS
3 months	69 (64;72)	66 (62;70)	71 (70;73)	*p* < 0.001	70 (65;74)	68 (60;72)	74 (68;77)	*p* = 0.010
6 months	67 (62;71)	63 (60;66)	71 (70;73)	*p* < 0.001	69 (63;72)	65 (59;70)	71 (70;74)	*p* = 0.018
12 months	64 (59;68)	60 (57;64)	68 (67;71)	*p* < 0.001	67 (61;69)	61 (68;71)	68 (66;72)	*p* = 0.020
HR % decrease								
3 months	17.8 (12.8;25)	22 (14;25)	15 (12;19)	*p* < 0.001	19 (11;24)	23.6 (13.2;25.3)	16.6 (10.6:20)	*p* < 0.001
6 months	20 (14;27)	24 (18;28)	13.6 (11;18)	*p* < 0.001	20 (13;27)	27.3 (16.5;28.8)	13.5 (11.2;20.8)	*p* < 0.001
12 months	21 (15;29)	26 (20;32)	15 (12;18)	*p* < 0.001	22 (16;28)	28.4 (17.7;30.4)	17.4 (15.4;25.4)	*p* < 0.001

Patients are stratified by their response to NSBB treatment according to outcome definition. Statistically significant differences are expressed by two-tailed *p* values. NSBB: non-selective beta-blocker, NS: not significant

In the derivation cohort group of non-responders (*N* = 43), six (13.95%) patients presented to the emergency department with variceal hemorrhage during the follow-up period, whereas the remaining 37 showed endoscopic upstaging of EVs grading. In the validation cohort group of non-responders (*N* = 12), two (16.66%) patients presented to the emergency department with variceal hemorrhage during the follow-up period, whereas the remaining ten showed endoscopic upstaging of EVs grading. In the derivation cohort of responders (*N* = 76), 56 (73.7%) patients showed downstaged EVs grading at the 12-month EGD, whereas 20 (26.3%) of patients showed stationary EVs grading at the 12-month EGD. In the validation cohort group of responders (*N* = 22), 13 (59.1%) patients showed downstaged EVs grading at the 12-month EGD, whereas 9 (40.9%) of patients showed stationary EVs grading at the 12-month EGD.

As reported in Table 2, median LS, HR, and SS values did not differ at enrollment evaluation between the responders and non-responders, their percentual decrease is statistically different across all follow-up appointments in both the derivation and validation cohort.

### Model analysis

According to the statistical analysis description reported in the methods section, model metrics are summarized in Table 3 for univariate models derived from our cohort of patients (i.e., percentual decrease in value after 3 months of NSBB therapy for SS, LS, and HR), univariate model adapted from Kim et al. [14] formula, and the combined model derived from our cohort of patients that incorporated the three variables of interest (i.e., SS, LS, and HR).

**Table 3:** Univariate and combined model metrics

Model metrics	Model 1 [Spleen stiffness]	Model 2 [Liver stiffness]	Model 3 [Heart rate]	Model 4 [Combined model]	Model 5 [Kim et al.]
Training cohort (*N* = 119)					
Intercept	4.78	0.96	1.7	5.6	0.0490
[95% C.I.]	[4.17;5.40]	[0.11;1.8]	[0.8;2.6]	[4.9;6.2]	
β-coefficients [95% C.I.]	− 0.55 [− 0.59; − 0.54]	− 0.16 [− 0.17; − 0.15]	− 0.13 [− 0.14; − 0.12]	Spleen stiffness − 0.43 [− 0.43; − 0.42] Liver stiffness − 0.16 [− 0.16; − 0.15] Heart rate − 0.02 [− 0.02; − 0.01]	Spleen stiffness * − 2.8345*
AUROC	0.89	0.78	0.72	0.96	0.88
Nagelkerke pseudo-*R*^2^	0.61	0.31	0.17	0.7	0.5
AIC	89.3	128.6	142.9	79.3	105.6
BIC	94.8	134.2	148.4	90.4	111.1
DeLong test (*p* value)	vs. Model 2: *p* = 0.020	vs. Model 1: *p* = 0.020	vs. Model 1: *p* < 0.001	vs. Model 1: 0.045	vs. Model 1: NS
	vs. Model 3: *p* < 0.001	vs. Model 3: NS	vs. Model 2: NS	vs. Model 2: *p* < 0.001	vs. Model 2: *p* = 0.036
	vs. Model 4: *p* = 0.045	vs. Model 4: *p* < 0.001	vs. Model 4: *p* < 0.001	vs. Model 3: *p* < 0.001	vs. Model 3: *p* < 0.001
	vs. Model 5: NS	vs. Model 5: *p* = 0.036	vs. Model 5: *p* < 0.001	vs. Model 5: *p* = 0.037	vs. Model 4: *p* = 0.037
Validation cohort (*N* = 34)					
AUROC	0.86	0.74	0.78	0.91	0.87

Models [1–4] are calculated on the study population and are referred to measurements in kPa, and the selected variables represent the percentual decrease at 3 months after therapy initiation. Intercept and β-Coefficients are provided by Kim et al. and applied to our cohort of patients to define discrimination and calibration. Values are logistic regression coefficients and 95% Confidence Interval (C.I.)

*AIC* Akaike information criterion, *BIC* Bayesian information criterion, *AUROC* area under the ROC-curve, Nagelkerke *R*^2^ pseudo-*R*^2^, *NS* not significant

As reported in Table 3, Model 4 showed statistically significant highest AUROC in both the training (0.96) and validation cohort (0.91), highest Nagelkerke Pseudo-R2 (0.70) and lowest AIC (79.3) and BIC (90.4) if compared to the univariate models derived from our cohort of patients and the univariate model derived from *Kim *et al*.* [14] formula.

For clarification purposes, to calculate the NSBB response probability using the combined model (i.e., Model 4), the following steps should be followed:

(i) obtain the percentual decrease of the variables at 3 months compared to index evaluation, and calculate the Linear Predictor (LP):

$${\text{LP}} = - 5.6 + \left( { + 0.43} \right) \times \left( {{\text{SS}}\% {\text{Decrease}}} \right) + \left( { + 0.16} \right) \times \left( {{\text{LS}}\% {\text{Decrease}}} \right) + \left( { + 0.02} \right) \times \left( {{\text{HR}}\% {\text{Decrease}}} \right)$$

(ii) Once the LP is obtained, the probability of being a responder can be calculated using the following formula:

$${\text{Probability}}\;{\text{of}}\;{\text{Being}}\;{\text{a}}\;{\text{Responder}}\;{\text{to}}\;{\text{NSBB}} = \frac{1}{{1 + e^{{ - {\text{LP}}}} }}$$

**Fig. 2:**
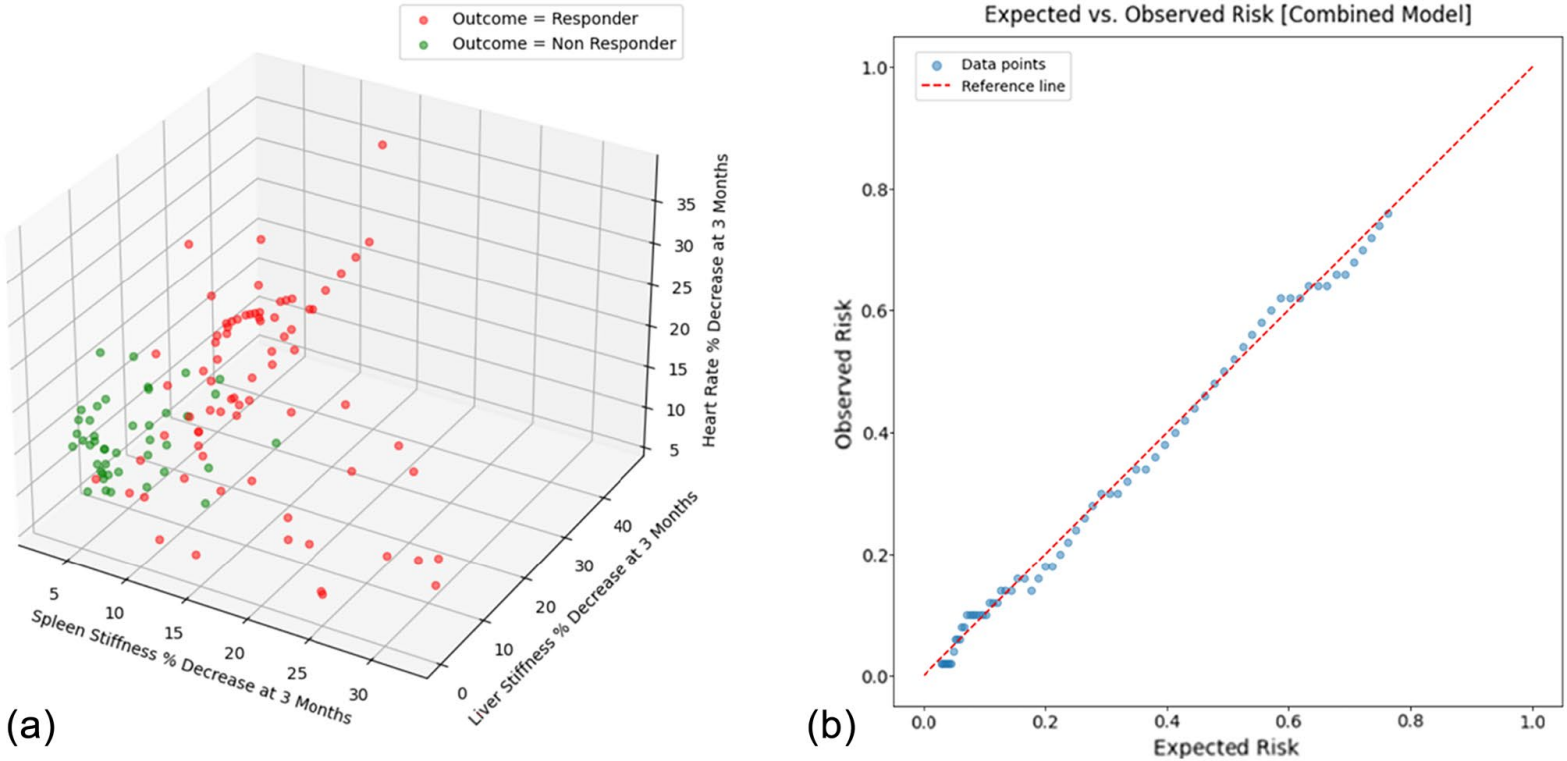
**a** Presents a 3D visualization of a logistic regression model, depicting the relationship between the predicted outcome and three input variables, offering a comprehensive view of the model’s decision boundary. **b** Displays the calibration plot comparing the expected versus observed risk for the model, evaluating how well the predicted probabilities align with actual outcomes

Model 4 is represented in a 3D graph in Fig. 2a, and the calibration plot for the derivation cohort (expected vs. observed risk) is illustrated in Fig. 2b.

### Cut-off analysis and online calculator

Cut-off values, chosen to maximize specificity, are reported in Table 4, along with their sensitivity, NPV, PPV, and accuracy. For clarification purposes, we converted the univariate model cut-offs into the respective variable percentual change at 3 months. In the training cohort, the cut-off chosen for the combined model was 0.90, which showed a statistically significant higher sensitivity (94.2%) if compared to SS (78.8%, *p* = 0.022), LS (23.1%, *p* < 0.001), and HR (22.1%, *p* < 0.001). The application of the same cut-off value in the validation cohort resulted in similar findings with a specificity of 97%, and a statistically significant higher sensitivity (93.8%) if compared to SS (76.5%, *p* = 0.018), LS (31.2%, *p* < 0.001), and HR (21.5%, *p* < 0.001). To make the tool available for clinicians, we have developed an online app (http://esophagealvarices.org) that allows for point-of-care entry of the variables of interest and automatic generation of the predicted probability according to patients data.

**Table 4:** Cutoff analysis of univariate and multivariate models

Model metrics	Model 1 [Spleen stiffness]	Model 2 [Liver stiffness]	Model 3 [Heart rate]	Model 4 [Combined model]
Training cohort (*N* = 119)				
Cutoff value	0.825 [ΔSS = − 11.5%]	0.85 [ΔLS = − 16.8%]	0.83 [ΔH*R = * − 25.3%]	0.90
Sensitivity	78.8 (70.6–85.2)	23.1 (16.4–31.5)	22.1 (10.6–50)	*94.2 (88.5–97.2)*
Specificity	100 (86.2–96)	100 (96.8–100)	100 (95.1–100)	100 (96.8–100)
NPV	89.1 (82.2–93.6)	62.3 (53.3–70.5)	56.4 (47.4–65)	95.7 (90.2–98.1)
PPV	100 (95.6–100)	100 (96.8–100)	100 (97.3–100)	100 (96.8–100)
Accuracy	86.4 (79.1–91.5)	66.1 (57.2–74)	56.8 (47.8–65.4)	97.5 (92.8–99.1)
McNemar test (*p* value)	vs. Model 2: *p* < 0.001	vs. Model 1: *p* < 0.001	vs. Model 1: *p* < 0.001	vs. Model 1: *p* = 0.022
	vs. Model 3: *p* < 0.001	vs. Model 3: NS	vs. Model 2: NS	vs. Model 2: *p* = *p* < 0.001
McNemar test	vs. Model 4: *p* = 0.022	vs. Model 4: *p* < 0.001	vs. Model 4: *p* < 0.001	vs. Model 3: *p* = *p* < 0.001
Validation cohort (*N* = 34)				
Sensitivity	76.5 (71.5–81.5)	31.2 (19.8–41.3)	21.5 (16.5–26.5)	93.8 (88.8–98.1)
Specificity	93 (88–98)	91 (86.1–95.3)	95 (91.1–97.1)	97 (92.1–98.3)
NPV	88.5 (83.5–93.5)	63.5 (58.5–68.5)	58.2 (53.2–63.2)	95.3 (90.3–97.1)
PPV	98.5 (93.5–99.1)	94 (91.5–96)	93.1 (90–95.6)	99.5 (94.5–100)
Accuracy	85.5 (80.5–90.5)	67.5 (62.5–72.5)	60.3 (53.8–64.2)	97.2 (92.2–99)
McNemar test (*p* value)	vs. Model 2: *p* < 0.001	vs. Model 1: *p* < 0.001	vs. Model 1: *p* < 0.001	vs. Model 1: *p* = 0.018
	vs. Model 3: *p* < 0.001	vs. Model 3: NS	vs. Model 2: NS	vs. Model 2: *p* = *p* < 0.001
McNemar test	vs. Model 4: *p* = 0.018	vs. Model 4: *p* < 0.001	vs. Model 4: *p* < 0.001	vs. Model 3: *p* = *p* < 0.001

As reported in the methods section, we selected cutoffs with a specificity of 100% to have low rates of false positives. For univariate models, cutoffs are reported as model output or converted back to percentual changes from baseline values. Cutoff sensitivities were compared using the McNemar Test

*NS* not significant

## Discussion

**Fig. 3:**
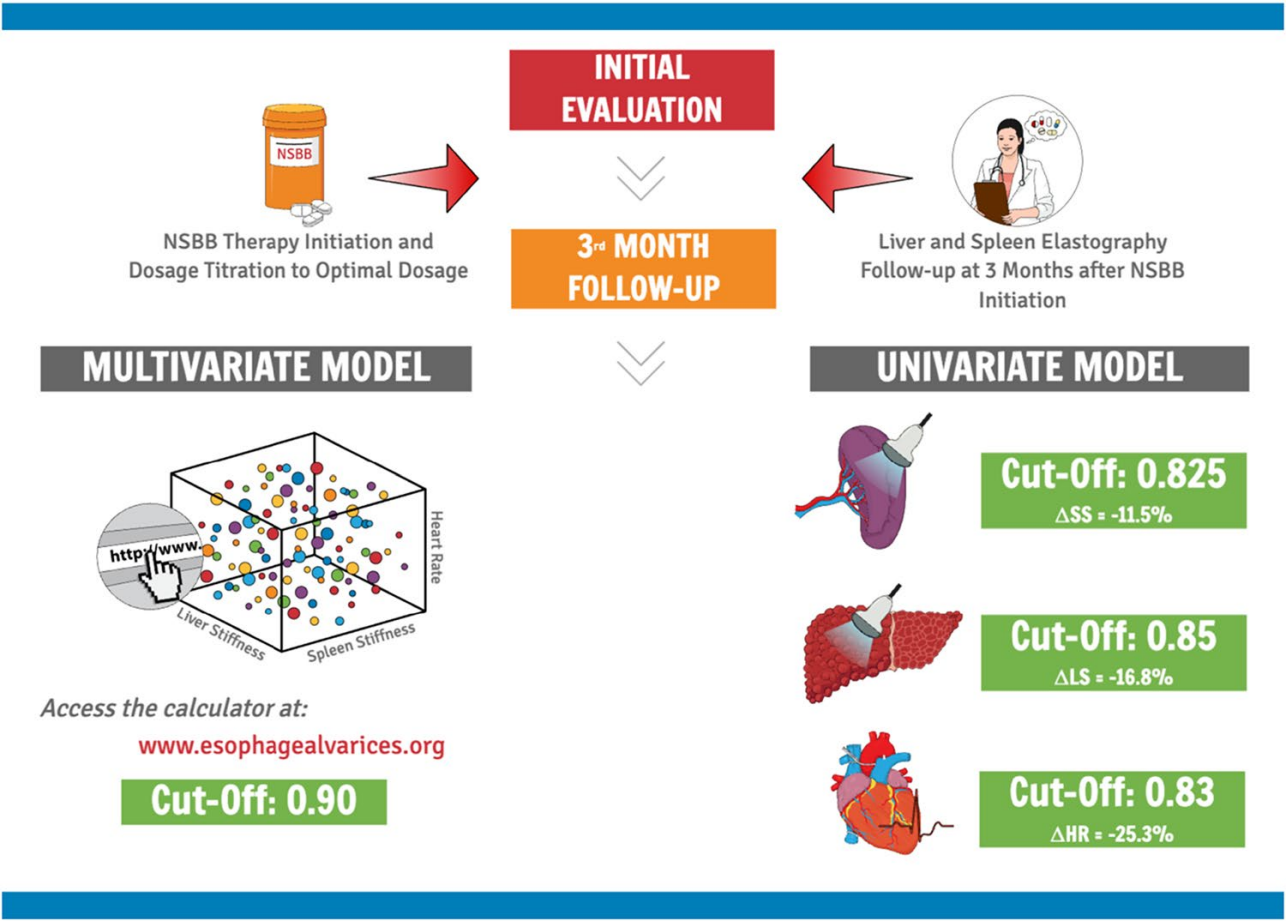
After initial evaluation and detection of HRVs, patients should be treated with NSBB if they present no contraindications. According to our findings, before NSBB therapy initiation LS, SS, and HR should be registered and repeated after 3 months to determine NSBB response non-invasively. Responses can be either evaluated by considering the multivariate model (1) or by considering each variable singularly (2)

The primary aim of this study was to assess the feasibility of using LS and SS changes to determine the therapeutic response to treatment with NSBB in patients with liver cirrhosis and documented presence of HRVs. The main results of this study indicate that across the initial included variables, only three showed a stronger signal in the outcome prediction. Those three variables were the percentage changes at the third month of follow-up evaluation for SS, LS, and HR and that, as shown in Fig. 3, according to their univariate analysis, a decrease > 11.5% for SS, > 16.8% for LS, and > 25.3% for HR were selected to have a specificity of 100%, in order to provide lower risks of detecting false positives (i.e., those patients who have not responded to NSBB, that were predicted as responders by the model). In terms of cut-off metrics in the univariate models, the SS percentual decrease showed a statistically significant higher sensitivity (78.8%), if compared to LS (31.2%, *p* < 0.001) and HR (21.5%, *p* < 0.001) in both the training and the validation set. Besides, the SS univariate model showed the highest discriminative ability (AUROC = 0.89) if compared to LS (AUROC = 0.78, DeLong’s Test—*p* = 0.020) or HR (AUROC = 0.72, DeLong’s Test—*p* < 0.001) and it showed the best calibration metrics (AIC = 89.3, BIC 94.8). The application of the model provided by Kim et al., showed no statistically significant differences in AUROC with SS (AUROC = 0.88 vs. AUROC = 0.89 respectively). At the same time, it showed, similarly to our univariate SS-based model, statistically significant difference in AUROC with LS and HR.

The multivariate model that incorporated SS, LS, and HR showed the highest discrimination in both the training (AUROC = 0.96) and validation (AUROC = 0.91), associated with the most performing calibration metrics (AIC = 79.3, BIC 90.4). We selected the cutoff value of 0.90, to maximize specificity up to 100%, which resulted also in a statistically significant higher sensitivity (94.2%), when compared to its univariate counterparts. The application of the model provided by Kim et al., showed no statistically significant differences in AUROC with our multivariate model (AUROC = 0.88 vs. AUROC = 0.96 respectively, DeLong’s Test – *p* = 0.037).

Our results on SS reflect those found by *Marasco *et al*.* [13]*,* who reported that changes in SS were linearly related to changes in HVPG (*r = *0.784, *p* value < 0.0001) and that changes in SS showed excellent discriminatory abilities (AUROC = 0.973), selecting a percentual decrease of -10% as the best cut-off to identify non-responders (sensitivity 100%, specificity 60%, NPV 100% and PPV 90%). In our study, the best SS cut-off to discriminate between responders and non-responders was a -11.5% at 3 months from the initial values. The authors also report that LS did not correlate with changes in HVPG (*r = *0.107, *p* value = 0.655), similar to what has been reported by *Binzberger *et al*.*[12]. In our study, however, the univariate analysis of LS percentual changes resulted in a model with a less discriminative ability and calibration if compared to SS, but still resulted in one of the three most critical features according to LASSO regression to predict NSBB response. *Rediberger *et al*.* [31] reported that the linear correlation between LS and HVPG measurement became stronger in patients under treatment with NSBB, which was higher in responders (*r = *0.864) than in non-responders (*r = *0.535). Therefore, regarding LS, the study by *Marasco *et al*.* [13] may not have found sufficient correlation due to the limited sample size and that LS is a significantly less valid predictor for NSBB response if considered univariately.

*Kim *et al*.* [14] developed a univariate model in a training cohort of 106 patients and validated it in an external validation cohort of 63 patients. Response to NSBB was defined as a decrease in HVPG values ≥ 20% of the baseline value or an absolute value of HVPG < 12 mmHg after dose titration. The timing between HVPG measurements was not standardized amongst all patients, and the authors did not provide any information about the calibration metrics of the model. Their model showed excellent discrimination metrics in the training (AUROC = 0.801) and the validation cohort (AUROC = 0.848). As mentioned above, the application of the model proposed by *Kim *et al*.* [14] on our cohort of patients and our univariate model based on SS showed comparable calibration metrics, slightly in favor of our approach, thus highlighting how a percentual decrease if compared to an absolute value change may slightly better reflects changes in splenoportal hemodynamic.

However, both the McNemar that the Delong test did not provide statistically significant differences between our univariate SS model and the one that *Kim *et al*. proposed* [14] regarding discrimination and cutoff sensitivity analysis. The remarkable similarity to our findings, on the one hand, enables us to serve as an external validation group for a study involving a patient cohort with diverse geographic backgrounds (Korean vs. Italian patients). In addition, this has allowed us to surmount the most substantial limitation of the study, specifically the lack of HVPG measurement at our center. Consequently, this provided enhanced rigor to our results and the methodologies employed in determining the clinical response to NSBB therapy.

The true novelty of the current study lies in developing a combined model that considers both splenic/hepatic elastography values and changes in heart rate (i.e., three known different non-invasive indicators of NSBB response), and that the combined model showed the highest AUROCs in both the derivation and validation cohorts and to readily understand how the probability of NSBB changes across variations of each of the three variables.

The multivariate model exhibits the most efficient metrics in both discrimination and calibration. As shown in Fig. 2b, it is noteworthy that the expected versus observed risk is nearly entirely superimposable on the reference line. From a statistical point of view, according to LASSO regression, the highest weight of the model was given by SS values (2.68-fold higher than LS and 21.5-fold higher than HR), thus implying that most of the estimates are determined by SS and that LS and HR changes play only a minor role in the outcome prediction, thus providing insight on the relative weight of NSBB-induced effects on each of these non-invasive methods widely used to define CSPH and eventually monitor NSBB response. Also, the relevance of the model lies in the fact that there is a general paucity of data on assessing the hemodynamic response to NSBBs for HRVs. Furthermore, the prophylaxis of the first bleeding event is associated with a number needed to treat of 5–13 patients, which means that many patients are undergoing therapy without an actual efficacy on the primary prevention of bleeding from HRVs, thereby exposing them to potential side effects. These results have been highlighted by a meta-analysis by *Zacharias *et al*.* [32], which evaluated carvedilol compared to other NSBBs without finding any difference in efficacy on primary prophylaxis or the onset of adverse events. However, significantly more deaths, episodes of upper gastrointestinal bleeding, and serious adverse events occurred in the long-term trials. Therefore, it is essential to continue NSBB treatment only for patients who achieve a hemodynamic response; currently, HVPG measurement is the sole method for evaluating the efficacy of NSBB treatment.

Our study is limited by the unavailability of HVPG measurements at our center, and the absence of an external validation cohort from a separate center. However, our cohort is the largest to date studying NSBB response with elastography with consistent follow-up, resulting in high-quality data collection for the duration of the study. We did have a temporally separated validation cohort where we documented similar results from *Kim *et al*.* [14], which suggests that our findings are externally applicable.

## Conclusions

Our study validates the result of *Kim *et al*.* [14] and defines a critical percentual decrease of SS in line with the findings of *Marasco *et al*.* [13], thus highlighting the potential role of spleen elastography in the prediction of NSBB response and as a practical ad non-invasive tool to monitor NSBB efficacy. Besides, the SS, LS, and HR combination provides a model with better prediction metrics than SS alone. Further studies are needed to validate our results and to verify if this technique can also be useful during the follow-up for monitoring portal pressure variations during the treatment and to possibly increase prediction capabilities of SS, LS, HR changes by reducing the re-evaluation intervals to the first 3 months after treatment initiation.
